# Identification of natural killer cell-related characteristics to predict the clinical prognosis and immune microenvironment of patients with low-grade glioma

**DOI:** 10.18632/aging.204850

**Published:** 2023-07-05

**Authors:** Fei Sun, Hongtao Lv, Baozhi Feng, Jiaao Sun, Linyun Zhang, Bin Dong

**Affiliations:** 1 Department of Neurosurgery, The First Affiliated Hospital of Dalian Medical University, Dalian, Liaoning, China; 2 Department of Neurosurgery, Xinhua Hospital Affiliated to Dalian University, Dalian, Liaoning, China; 3 Department of Urology, The First Affiliated Hospital of Dalian Medical University, Dalian, Liaoning, China

**Keywords:** low-grade glioma (LGG), natural killer (NK) cells, tumor immune microenvironment, RiskScore model, bioinformatics

## Abstract

Background: Individuals with low-grade glioma (LGG) have a dismal prognosis, and most patients will eventually progress to high-grade disease. Therefore, it is crucial to accurately determine their prognoses.

Methods: Seventy-nine NK cell genes were downloaded from the LM22 database and univariate Cox regression analysis was utilized to detect NK cell-related genes affecting prognosis. Molecular types were established for LGG using the “ConsensusClusterPlus” R package. The results from a functional enrichment analysis and the immune microenvironment were intensively explored to determine molecular heterogeneity and immune characteristics across distinct subtypes. Furthermore, a RiskScore model was developed and verified using expression profiles of NK cells, and a nomogram consisting of the RiskScore model and clinical traits was constructed. Moreover, pan-cancer traits of NK cells were also investigated.

Results: The C1 subtype included the greatest amount of immune infiltration and the poorest prognosis among well-established subtypes. The majority of enriched pathways were those involved in tumor progression, including epithelial-mesenchymal transition and cell cycle pathways. Differentially expressed genes among distinct subtypes were determined and used to develop a novel RiskScore model. This model was able to distinguish low-risk patients with LGG from those with high-risk disease. An accurate nomogram including the RiskScore, disease grade and patient’s age was constructed to predict clinical outcomes of LGG patients. Finally, a pan-cancer analysis further highlighted the crucial roles of NK cell-related genes in the tumor microenvironment.

Conclusions: An NK cell-related RiskScore model can accurately predict the prognoses of patients with LGG and provide valuable insights into personalized medicine.

## INTRODUCTION

Brain tumors are the tenth most fatal human malignancy, accounting for around 3 percent of all cancer deaths, according to recent data from cancer centers [1]. Glioma is the most common malignant intracranial tumor [2, 3]. The World Health Organization (WHO) has classified gliomas by grades based on their pathological characteristics, with grades I and II representing low-grade glioma (LGG) and grades III and IV representing high-grade glioma (HGG). LGG accounts for approximately 15% of all gliomas. Most LGGs eventually progress to HGGs, with poor prognosis [4]. At present, LGG tumors are often surgically resected, accompanied by radiotherapy and chemotherapy [5, 6]. Due to the invasive growth characteristics of gliomas, surgical resection or radiotherapy cannot avoid tumor recurrence [7]. Therefore, it is necessary to determine an efficient treatment plan. Currently, when treating LGG, it is best to surgically remove as much of the tumor as possible, but the treatment plan for individuals who have unresectable disease is highly controversial. Therefore, an in-depth understanding of the pathogenesis of LGG is essential to manage and treat patients with LGG to improve their prognoses.

In order for cancer cells to form and spread, they need a specific environment known as the tumor microenvironment (TME) [8, 9]. The infiltration of immune cells into the TME has been shown to influence cancer progression and has strong prognostic value for LGG [10]. The onset and progression of LGG are inextricably linked to the dysfunction of the body’s immune system, particularly natural killer (NK) cells, which perform non-specific immune responses [11]. These cells preferentially eliminate major histocompatibility complex class I downregulated neoplastic tumors [12]. During homeostasis, NK cells reside in the brain parenchyma and circulate through the blood-brain barrier (BBB) [13]. The presence of NK cells in brain tumors and the surrounding brain parenchyma microenvironment has been previously identified [14, 15]. It is unclear, however, which factors facilitate NK cell passage across the BBB and subsequent activation in the brain. Further evidence suggests that *NKp46*, *NKp44*, and *NKp3* can synergistically inhibit or activate the antitumor function of NK cells [16]. In addition, NK cells recognize a variety of cell- and tumor-associated surface ligands and may control tumor cell function in a positive or negative manner [17]. *In vitro*, glioma cell lines can be killed by NK cells [18]. LGG is a highly vascularized tumor that can alter the immune system and impair immune system function [19]. The relationship between NK cells and LGG is still not completely understood. Hence, for the development of new immunotherapies, understanding immune surveillance mechanisms in LGG patients with greater survival rates is critical.

Through using the Chinese Glioma Genome Atlas (CGGA) and The Cancer Genome Atlas (TCGA) databases [20], this study carried out molecular typing of LGG based on LM22 data and transcriptomic data using NK cell-related genes [21]. In addition, a risk model was constructed, and immune cells in the LGG tumor microenvironment (TME) were also assessed. Discrepancies in drug sensitivity among different clusters were also predicted. Immune profiles and therapeutic sensitivities significantly differed among LGG molecular subtypes. Finally, this study summarized a pan-cancer overview of NK cell-related genes, thereby elucidating the relationship between LGG and NK cells, and informing the personalized treatment of LGG.

## METHODS

### Data acquisition and processing

Similar to the method used in previous studies [22, 23], clinical samples and mRNA transcriptome data of LGG were accessed using the CGGA (http://www.cgga.org.cn/) and TGCA (https://portal.gdc.cancer.gov/) databases. Exclusion criteria were as follows: samples with incomplete clinical follow-up data including survival time and status. Two datasets, “mRNAseq_325 (batch 2)” and “mRNAseq_693 (batch 1)”, from the CGGA were merged using the “RTCGAToolbox” package and named as the “CGGA dataset”. Subsequently, batch effects were eliminated using the “sva” package. Clinical information of the TCGA and CGGA datasets is shown in Supplementary Table 1. Gene mutation and DNA methylation data of LGG patients were downloaded from the TCGA database [24, 25]. Transcriptome data for NK cell-related genes, containing 79 NK cell genes, were downloaded from the LM22 database (Supplementary Table 2) [21]. The LM22 database contains 547 genes that distinguish 22 human hematopoietic cell phenotypes, including T cells, naïve and memory B cells, plasma cells, NK cells, and myeloid subsets.

### Molecular typing

Univariate Cox regression analysis was implemented to determine NK cell genes associated with LGG prognoses in the TCGA and CGGA datasets. The LGG molecular subtypes were obtained using the “ConsensusClusterPlus” R package based on prognosis-related NK cell genes [26]. In total, 500 bootstraps were conducted using the “K-M” algorithm and “canberra” as the metric distance, with 80% and 20% of patients in the respective training and validation sets involved in each bootstrapping process. Clusters ranged from 10 to 2, and molecular subtypes were determined using the optimum classification system established by calculating the consistency cumulative distribution function (CDF) and the consistency matrix [27].

In 2020, Zheng et al. stated that patients with LGG were categorized into six molecular subtypes, including Codel, G-CIMP-high, G-CIMP-low, Classic-like, Mesenchymal-like and PA-like [28]. To comprehensively uncover the molecular traits of our NK cell-based typing, we also investigated the close association of NK cell subtypes with these six molecular subtypes.

### Gene set enrichment analysis (GSEA)

The optimal molecular subtyping was determined, and subsequent differential gene analysis was performed on the different LGG molecular types using the “limma” package with thresholds set at *P* < 0.05 and |log fold change (FC)| > 1 [29]. Candidate gene sets in The Molecular Signatures Database (MSigDB) (https://ngdc.cncb.ac.cn/databasecommons/database/id/1077) [30] were subjected to “GSEA” enrichment analysis to explore possible pathways linked to DEGs to screen significant pathways using a FDR < 0.05 [30]. In addition, gene sets related to angiogenesis and inflammatory characteristics were collected from previously published studies [31, 32].

### Tumor immune microenvironment and clinical characteristics in different clusters

The following immune prediction algorithms were utilized to explore discrepancies in the tumor immune microenvironment among distinct clusters: “CIBERSOFT” and “ESTIMATE” algorithms. The “CIBERSOFT” algorithm assessed the infiltration abundance of various immunocytes in the TCGA and CGGA datasets, while the “ESTIMATE” algorithm calculated the “ImmuneScore”. The “ImmuneScore” reflects the cumulative content of immune cells in the TME. Importantly, immune-associated signaling pathways were also explored, including interferon and MHC.

The TCGA and CGGA datasets provided a series of clinical information, including age, grade, and IDH mutation. These indicators are key factors in clinical decision-making and therapeutic strategies for LGG patients. Moreover, discrepancies in clinical information among these four clusters were also investigated.

### Immunotherapy/chemotherapy prediction in different clusters

A recent study by Palmeri et al. stated that the tumor mutational burden (TMB) could serve as an accurate biomarker for immunotherapy outcome prediction [33]. Davoli and colleges further reported that tumor aneuploidy could also be used as an immunotherapy biomarker [34]. Thus, we compared TMB levels and aneuploidy scores among different clusters. Importantly, the expression of various typical immune checkpoint genes among different clusters was also explored. The IC50 of chemotherapy drugs in the GDSC (Genomics of Drug Sensitivity in Cancer) database, which contained a large panel of cancer cell lines, was calculated using the “pRRophetic” package [35]. Finally, IC50 values of each drug among different clusters were also compared.

### Risk model construction

The “Limma” package was implemented to determine differentially expressed genes (DEGs) among different clusters. Subsequently, these DEGs were selected to develop a risk model using least absolute shrinkage and selection operator (LASSO) and multivariate Cox regression analysis. Specifically, the number of model genes was reduced using a LASSO regression to further screen the valid genes. LASSO regression solved the multicollinearity during the regression study by compressing coefficients and setting some of them to zero. Alongside the gradual increase in lambda, selection of the optimum number of factors was carried out when the coefficients of independent variables tended to zero. In addition, the best model was developed by performing stepwise regression applying the Akaike information criterion (AIC) in the “MASS package”. This process of regression takes into consideration the best statistical fit of the model and parameter number. Model development was conducted by initially establishing the most complex model, followed by a successive reduction in the number of variables involved to decrease the AIC values. The lower the AIC values, the better the model, as these low values indicated the least number of parameters used to achieve a sufficient model fit. After determining valid genes, a risk model was constructed by the following equation: RiskScore = Exp_i_ × Σβ_i_, where β is the multivariate Cox regression coefficient of a corresponding gene, and Exp_i_ refers to the expression of NK cell-related gene characteristics [36]. Subsequently, a z-score was obtained, and patients were classified into low-RiskScore and high-RiskScore groups with the criteria of z-score = 0. Furthermore, the Kaplan-Meier method was used to plot survival curves for prognostic analysis. Moreover, the log-rank test was performed to examine whether differences were considered significant.

### Immune microenvironment, molecular characteristics, and drug prediction in the risk model

In LGG, the CIBERSORT (https://cibersort.stanford.edu/) algorithm calculated the relative abundance of 22 immune cells to analyze the differences in immune cell composition and function between high- and low-RiskScore groups. Additionally, to measure immune cell amounts, the “ESTIMATE” R package was applied to calculate the ImmuneScore, the StromalScore and the ESTIMATEScore [37]. Based on previous studies, scores of 10 oncogenic pathways (cell cycle, HIPPO, MYC, NOTCH, NRF1, PI3K, TGF-Beta, RAS, TP53, WNT), 7 metagenes pathways (HCK, IgG, Interferon, LCK, MHC_I, MHC_II, STAT1), T cell inflamed GEP, and cytolytic activity were calculated by “ssGSEA” using the “GSVA” package [38]. Discrepancies in the above pathway activities between different risk groups were comprehensively investigated. Based on the results of the “pRRophetic” package, chemotherapy drug sensitivities between high- and low-RiskScore groups were also further explored.

### Nomogram

Univariate and multivariate Cox regression analysis using clinicopathological variables were performed to determine if the RiskScore may be used as an independent prediction measure [39]. The ‘rms’ software was used to construct a nomogram containing all independent prognostic factors to estimate 1-, 3-, and 5-year overall survival (OS) probability. The nomogram's discriminative power was measured using the concordance index (C-index) and calibration.

### Pan-cancer characterization of NK cell-related genes

Based on the pan-cancer cohort samples of the TCGA, we gathered and compiled genomic data, transcriptome data, and clinical follow-up information for dozens of human malignancies. It is currently recognized that information on promoter methylation levels and gene mutations may have a significant effect on gene expression. Hence, we first investigated the pan-tumor mutation and methylation data of NK cell-related genes. Of note, we interpreted the mutation spectrum primarily from the viewpoints of CNV and SNV. Subsequently, we conducted a complete analysis of the differential expression of each NK cell-related gene in distinct malignant and precancerous tissues and evaluated the prognostic relevance of each gene across tumor types. Importantly, based on the Msigdb platform, we collected the classical metabolic pathways and immune pathways and, using the GSEA concept, and evaluated the potential correlation between the NK cell gene set and these pathways, thereby laying the groundwork for subsequent mechanistic studies.

### Statistical analysis

Data analysis was conducted using the R 4.0.2 statistical tool (https://www.r-project.org/). The “limma” package was used for group differential gene analysis, the “MASS package” for stepAIC analysis, the “ssGSEA” for enrichment pathway analysis, and the “survminer” package for survival analysis. Furthermore, the examination and comparison of survival differences between the aforementioned groups were carried out using the Kaplan-Meier (K-M) method and the log-rank test. The “rpart” and “rpart.plot” packages were utilized to develop a survival decision tree. Statistical tests were two-sided unless otherwise specified, and a significant difference was indicated by a *P* value < 0.05.

### Availability of data and materials

The datasets analyzed in this work may be found in the Supplementary Materials or contact with the corresponding author.

## RESULTS

### Molecular typing based on NK cell-related genes

**Figure 1:**
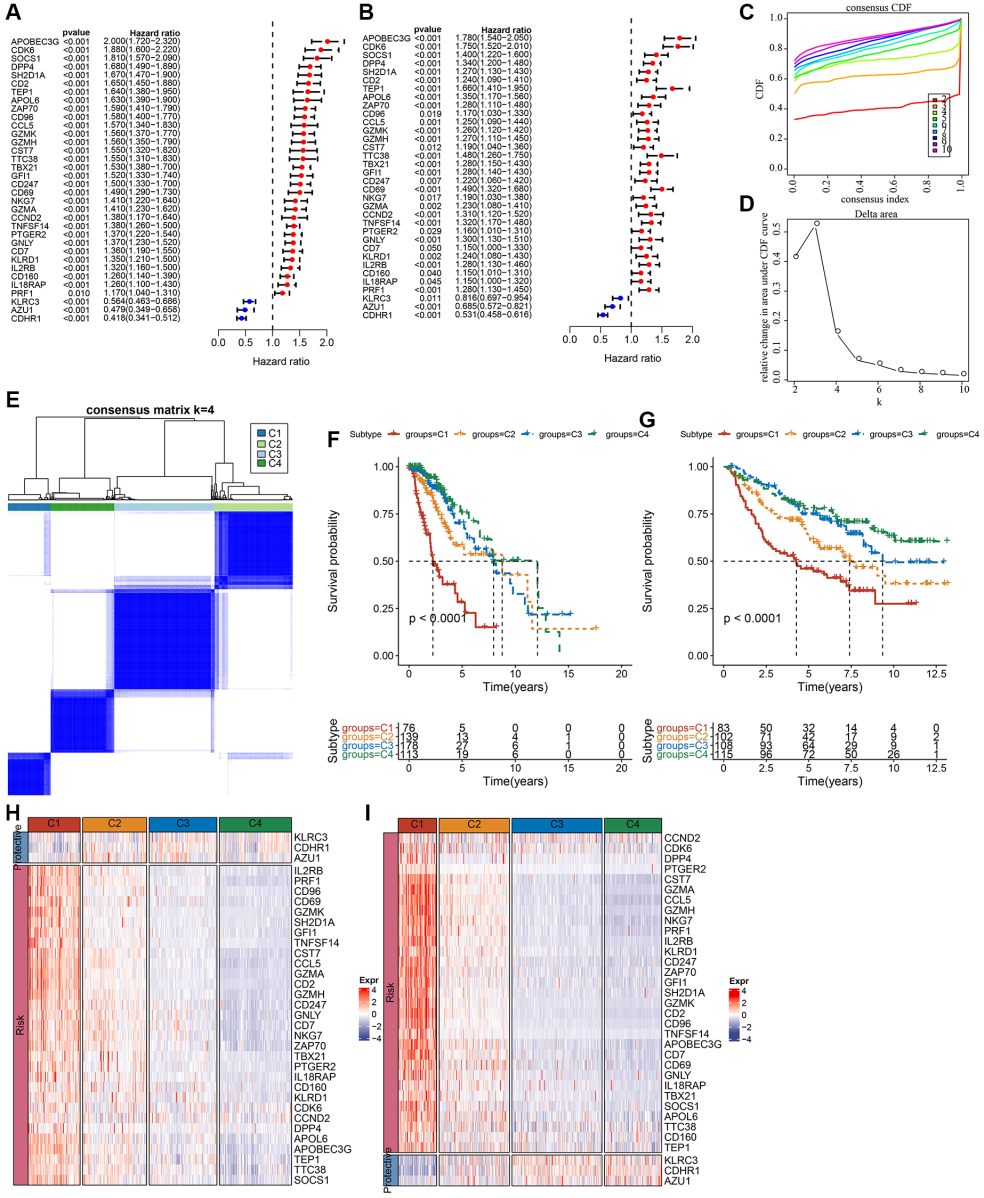
**Molecular subtyping of LGG based on NK cell-related genes.** (**A**) Univariate Cox forest plot of NK cell-related genes in the TCGA-LGG cohort. (**B**) Multivariate Cox forest plot of NK cell-related genes in the CGGA-LGG cohort. (**C**) CDF curve of samples in the TCGA-LGG cohort. (**D**) CDF delta area curve of TCGA-LGG cohort samples area curve, Delta area curve of consensus clustering indicated the relative variation in the area under the CDF curve for each category number k in comparison with k−1. The vertical axis represents the relative change in area under the CDF curve, the horizontal axis represents the category number k. (**E**) A heat map of sample clustering at consensus k = 4. (**F**) K-M survival curves showing the prognosis of the four subtypes in the TCGA-LGG cohort. (**G**) K-M survival curves for the prognosis of the four subtypes in the CGGA cohort. (**H**) A heat map showing the expression of prognostically significant NK cell-related genes in various subtypes in TCGA-LGG. (**I**) A heat map of the expression of prognostically significant NK cell-associated genes in various subtypes in CGGA. Abbreviations: CDF: cumulative distribution function; expr: expression; LGG: low-grade glioma; NK: natural killer; TCGA: The Cancer Genome Atlas; CGGA: Chinese Glioma Genome Atlas; K-M: Kaplan-Meier.

LGG-related prognostic genes in the TCGA and CGGA datasets were analyzed separately using univariate Cox regression. Genes associated with NK cells were examined for correlation with OS in individuals with LGG, which revealed a link between 42 genes in the CGGA dataset (*P* < 0.05) and 51 out of 79 genes in the TCGA dataset (*P* < 0.05). Significant prognostic genes are displayed in Supplementary Table 3. Subsequently, prognostic genes in both datasets were intersected to obtain 34 prognosis-related significant genes (Figure 1A, 1B). Subsequently, the optimum number of clusters in the TCGA dataset was identified using consensus clustering for the aforementioned prognostic-related genes. Cluster number was defined according to CDF, which was reflected by the CDF delta area curves, and was determined as a stable cluster when the number of clusters was 4 (Figure 1C, 1D). Therefore, clustering was optimal when k = 4 (Figure 1E). Clusters of each LGG in the TCGA and CGGA datasets are displayed in Supplementary Table 4. Survival and K-M curve analysis suggested a better prognosis for cluster C4, whereas cluster C1 had the worst prognosis (Figure 1F). Similarly, LGG subtypes in the CGGA dataset were classified into four categories (k = 4), and the survival curves for each subtype were examined (Figure 1G). Variations between the expression of 34 NK cell-linked genes among various subtypes were compared. In the two independent datasets, risk genes exhibited increased expression in the C1 cluster, whereas protective genes depicted increased expression in the C4 cluster (Figure 1H, 1I).

### Genomic landscape and pathway characteristics among molecular subtypes

In the TCGA-LGG cohort, molecular characteristics were compared among the four subtypes to investigate the differences in genomic alterations among them. Analysis revealed that the C1 cluster had a high TMB score and a high aneuploidy score (Supplementary Figure 1A). In addition, six previously established molecular subtypes [28] were compared with the currently defined four molecular subtypes. The results highlighted more “G-CIMP-high” and “Codel” molecular subtypes in clusters C3 and C4 (Supplementary Figure 1B). Subsequently, significant mutation differences among subtypes were determined. After visualizing the top 20 mutated genes, mutation frequencies of gene, such as isocitrate dehydrogenase (*IDH*)*1* and capicua (*CIC*), varied considerably among the four molecular subtypes (Supplementary Figure 1C).

Additionally, among the four subtypes, a distribution comparison of clinical characteristics was carried out in the TCGA dataset (Supplementary Figure 2A), and no significant difference was found between genders. A larger proportion of patients with the C1 subtype had a G3 grade and more patients in subtypes C3 and C4 had a G2 grade. As for *IDH* mutations, the highest proportion of patients that had mutations were in subtypes C4, C3, and C2. In addition, the majority of patients in subtypes C4 and C3 had IDH mutations and *IDH*mut-non-codel. O-6-methylguanine-DNA methyltransferase (MGMT) promoter methylation level was also considerably increased in C3 and C4 subtypes rather than in C1 (Supplementary Figure 2A). Age, MGMT promoter methylation, *IDH* mutation, disease grade, and 1p19q co-deletion in the CGGA dataset were also examined (Supplementary Figure 2B). Gender did not show a significant difference among subtypes, while the 1p19q co-deletion level and *IDH* mutation were considerably increased in C3 and C4 subtypes when compared to C1, which was similar to the observations reported in the above analysis of the TCGA dataset.

**Figure 2:**
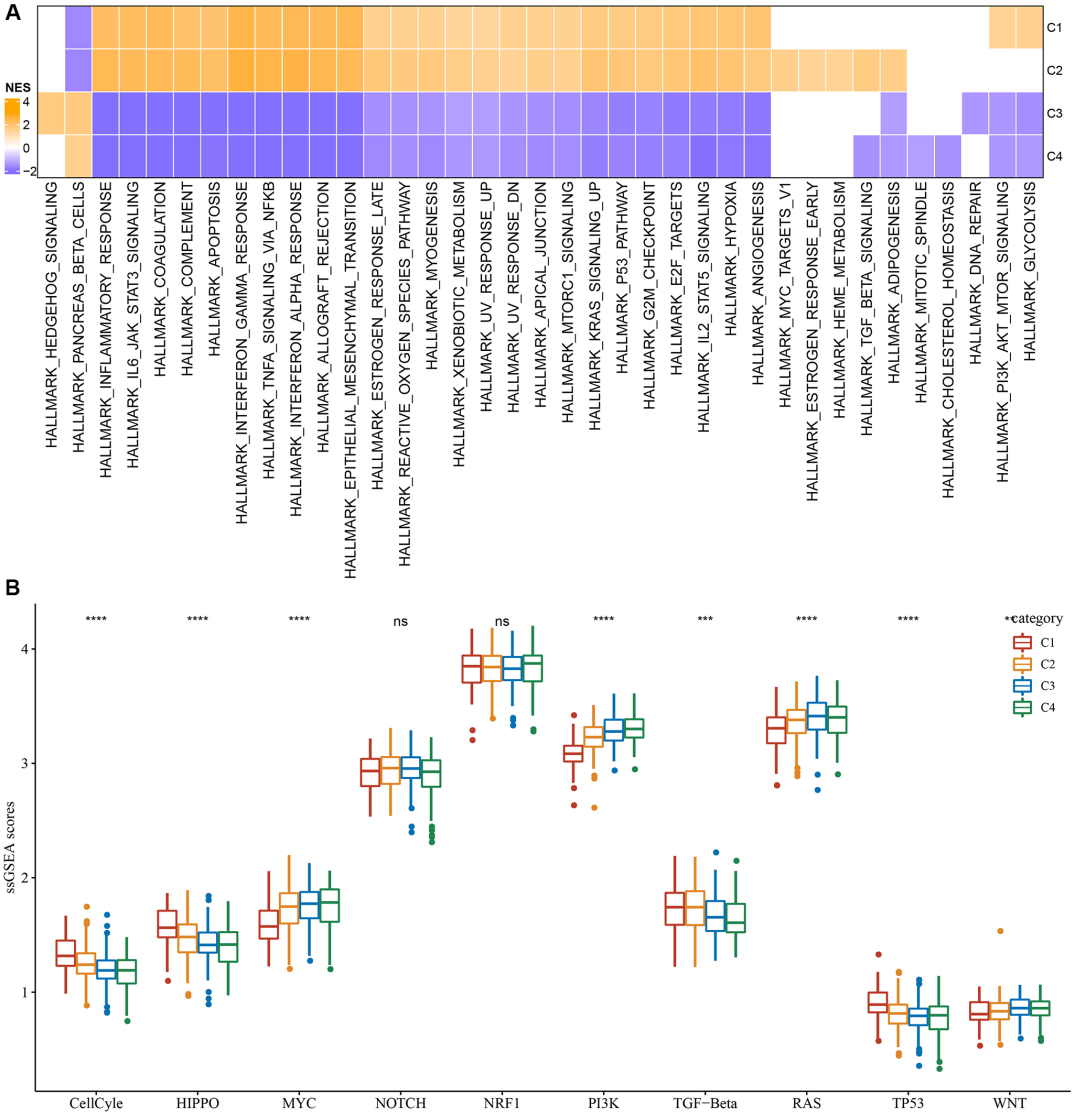
**Significantly activated pathways in different molecular subtypes.** (**A**) GSEA analysis results in the TCGA-LGG cohort. (**B**) Variation in the scores of 10 tumor abnormality-related pathways in various TCGA-LGG molecular subtypes in the ^**^P < 0.01; ^***^P < 0.001; ^****^P < 0.0001. Abbreviations: ns: no significance; NES: normalized enrichment scores; ssGSEA: single-sample GSEA; GSEA: gene set enrichment analysis; TCGA: The Cancer Genome Atlas; LGG: low-grade glioma; TP53: tumor protein p53; PI3K: phosphatidylinositol 3-kinase; NRF1: nuclear respiratory factor-1; TGF-β: transforming growth factor-β.

Furthermore, differentially activated pathways among subtypes were categorized by the “GSEA” enrichment analysis. Findings based on the TCGA cohort suggested considerable enrichment of cancer-associated pathways in subtypes C1 and C2, such as glycolysis, PI3K-AKT-mTOR, angiogenesis, hypoxia, P53, and apoptosis (Figure 2A). Moreover, the cell cycle, HIPPO, and TP53 pathway activities were also noticeably upregulated in the C1 subtype (Figure 2B). In short, we found that several signaling pathways were closely related to tumorigenesis and development which showed a significantly up-regulated trend in the C1 and C2 subtypes, and a significant downward trend in the C3 and C4 subtypes. This phenomenon may be potentially contributing to the observed difference in prognosis.

### Differences in immunological characteristics and treatment efficacy among molecular subtypes

**Figure 3:**
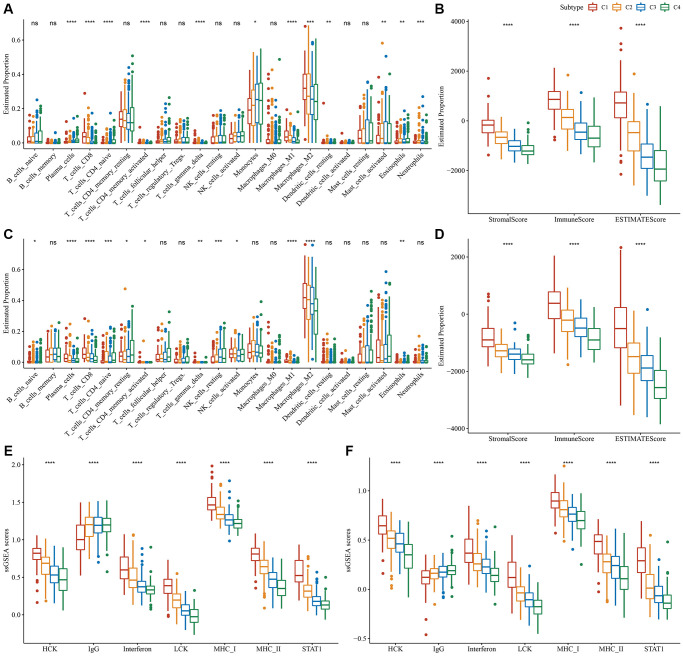
**Degree of immune cell infiltration in different molecular subtypes.** (**A**) Variation in 22 immune cell scores among various TCGA-LGG molecular subtypes. (**B**) Variation in ESTIMATE immune infiltration among various TCGA-LGG molecular subtypes. (**C**) Variation in 22 immune cell scores among different CGGA cohort molecular subtypes. (**D**) Variation in ESTIMATE immune infiltration among CGGA cohort molecular subtypes. (**E**) Variation in the gene cluster scores for the seven inflammation-related genesets among molecular subtypes in the TCGA-LGG cohort. (**F**) Differences in the gene cluster scores for the seven inflammation-related genesets among molecular subtypes in the CGGA cohort. ^*^P < 0.05; ^**^P < 0.01; ^***^P < 0.001; ^****^P < 0.0001. Abbreviations: ns: no significance; ssGSEA: single-sample GSEA; TCGA: The Cancer Genome Atlas; GSEA: gene set enrichment analysis; CGGA: Chinese Glioma Genome Atlas; LGG: low-grade glioma; HCK: hematopoietic cell kinase; IgG: Immunoglobulin G; LCK: lymphocyte-specific protein tyrosine kinase; MCH: melanin-concentrating hormone; STAT1: signal transducer and activator of transcription 1.

Variations in the immune microenvironment among subtypes was analyzed by determining the relative frequency of the 22 immune cells using “CIBERSORT”. Most immune cells differed significantly among subtypes. For example, the C1 subtype had a higher infiltration of CD8^+^T cells and macrophages in the TCGA dataset. Additionally, the ESTIMATE algorithm revealed that the C1 subtype had the highest “ImmuneScore” and “StromalScore”, followed by the C2, C3, and C4 subtypes (Figure 3A, 3B). Importantly, similar findings were also observed in the CGGA dataset (Figure 3C, 3D). In addition, we analyzed the inflammatory activity of four molecular subtypes, and the enrichment scores of all the metagene clusters (i.e., HCK, IgG, Interferon, LCK, MHC-I, MHC-II, STAT1) were significantly different among the four molecular subtypes. Overall, the C1 subtype had higher inflammatory activity, as shown in Figure 3E; similarly, this phenomenon was also observed in the CGGA cohorts, as per Figure 3F.

**Figure 4:**
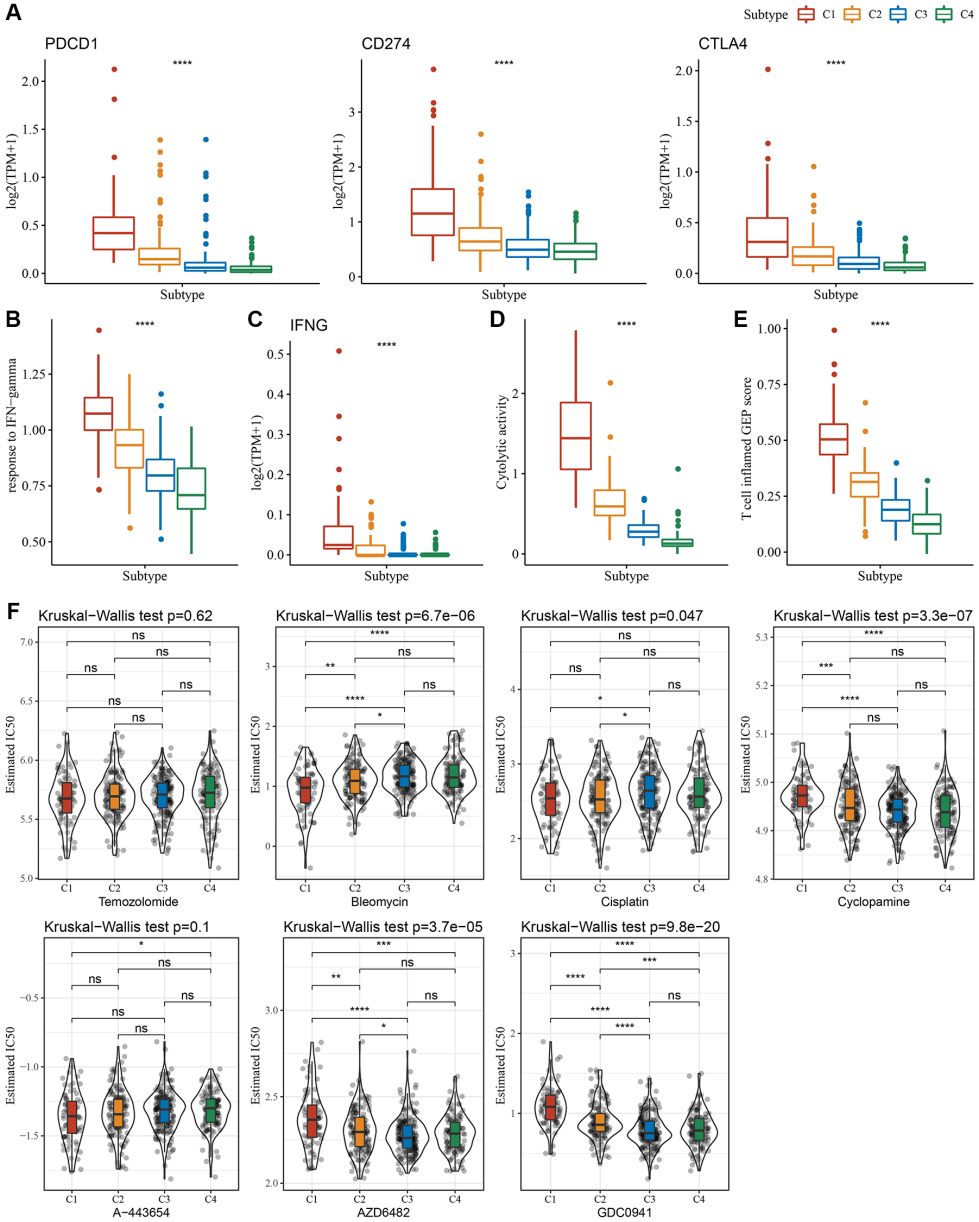
**Immunological characteristics scores characterizing the effect of immunotherapy in different subtypes.** (**A**) Expression differences of immune checkpoint-related genes among molecular subtypes. (**B**) Variation in response to IFN-γ among molecular subtypes. (**C**) The difference in expression of IFNG gene among molecular subtypes. (**D**) Differences in “Cytolytic activity” among molecular subtypes. (**E**) Variation in the T cell inflamed GEP score among molecular subtypes. (**F**) A box plot of the estimated IC_50_ values for temozolomide, bleomycin, cisplatin, cyclopamine, A-443654, AZD6482, and GDC0941 in TCGA-LGG. ^*^P < 0.05; ^**^P < 0.01; ^***^P < 0.001; ^****^P < 0.0001. Abbreviations: ns: no significance; TPM: transcripts per million; IFN: interferon; GEP: gene expression profile; IC_50_: half-maximal inhibitory concentration; TCGA: The Cancer Genome Atlas; LGG: low-grade glioma.

Since immune cell checkpoints are key targets for cancer treatment using immune checkpoint blockade (ICB), several checkpoint molecules, including cytotoxic T-lymphocyte-associated protein 4 (*CTLA-4*), programmed death 1 (*PD-1*), and programmed cell death-ligand 1 (*PD-L1*) were evaluated. These molecules showed significantly high expression in subtype C1 (Figure 4A). Given that IFN-γ plays a key role in immune modulation and anticancer immunity, we carried out ssGSEA analysis of the GOBP_RESPONSE_TO_INTERFERON_GAMMA gene set loaded from the GO database and found that the IFN-γ response is significantly enhanced in the C1 subtype (Figure 4B). Simultaneously, we also compared differences in the expression of the IFNG gene among the four subtypes and found that IFNG was significantly higher in the C1 subtype (Figure 4C). In addition, the cytotoxic (CYT) score, which characterized cytotoxic activity, was considerably increased in the C1 subtype (Figure 4D). In addition, we used the “T-cell-inflamed score” to evaluate the predictive potential of different molecular subtypes in cancer immunotherapy. As depicted in Figure 4E, the C1 subtype showed a higher T-cell-inflamed score. Taken together, these findings suggested that LGG patients with subtype C1 might benefit from immunotherapy.

Moreover, we analyzed the response of different molecular subtypes in the TCGA-LGG cohort to traditional chemotherapeutic drugs. C1 subtype patients were more sensitive to bleomycin, as evidenced by the biochemical half-maximal inhibitory concentration (IC_50_) and their response (Figure 4F).

### Establishment and validation of a clinical prognostic model

**Figure 5:**
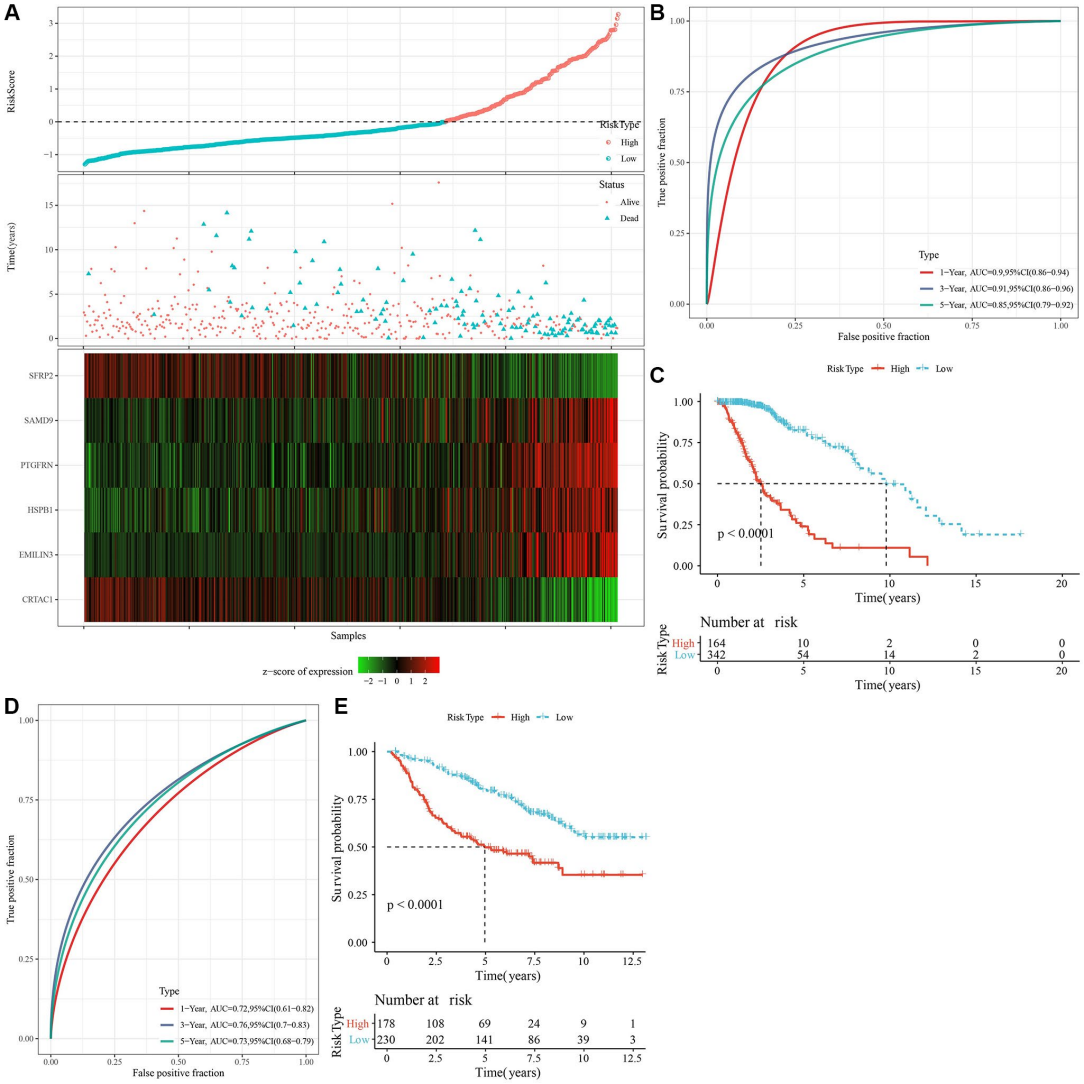
**Calculation of RiskScore and determination of its robustness by ROC.** (**A**) The RiskScore, survival status, survival time, and expression of oxidative stress-related prognostic genes in the TCGA dataset. (**B**) The ROC curve and AUC of RiskScore classification in the TCGA dataset. (**C**) The K-M survival curve distribution of RiskScore in the TCGA dataset. (**D**, **E**) The ROC curve and K-M survival curve of RiskScore in the CGGA cohort. Abbreviations: AUC: area under the ROC curve; CI: confidence interval; ROC: receiver operating characteristic; TCGA: The Cancer Genome Atlas; K-M: Kaplan-Meier; CGGA: Chinese Glioma Genome Atlas.

We identified a total of 739 DEGs among C1 vs. other, C2 vs. other, C3 vs. other, and C4 vs. other using the limma analysis (Supplementary Figure 3A–3C). There were no significant differentially expressed genes between C2 and other subtypes. Subsequently, the prognostic performances of the above 739 DEGs were further explored, with the results showing that a total of 719 genes (including 466 “risk” and 253 “protective” genes) were significantly associated with LGG prognoses (Supplementary Figure 4A, Supplementary Table 5, *P* < 0.001). LASSO and multivariate Cox regression algorithms were utilized to construct a NK cell-based RiskScore. LASSO regression highlighted the crucial roles of 14 genes in the prognostic model (Supplementary Figure 4B, 4C). Finally, multivariate Cox regression analysis identified 6 genes to calculate the RiskScore (Supplementary Figure 4D). Using the z-score value = 0 as the dividing line, LGG patients were divided into high-risk group and low-risk groups, and the expression and distribution of model genes in each LGG patient were characterized (Figure 5A). For the TCGA and CGGA cohorts, KM survival analysis and ROC curves validated the accurate prediction performances of the RiskScore (Figure 5B–5E). Specifically, LGG patients with high RiskScore were associated with worse clinical outcomes.

### Association of the RiskScore with different clinicopathological features and molecular subtypes

**Figure 6:**
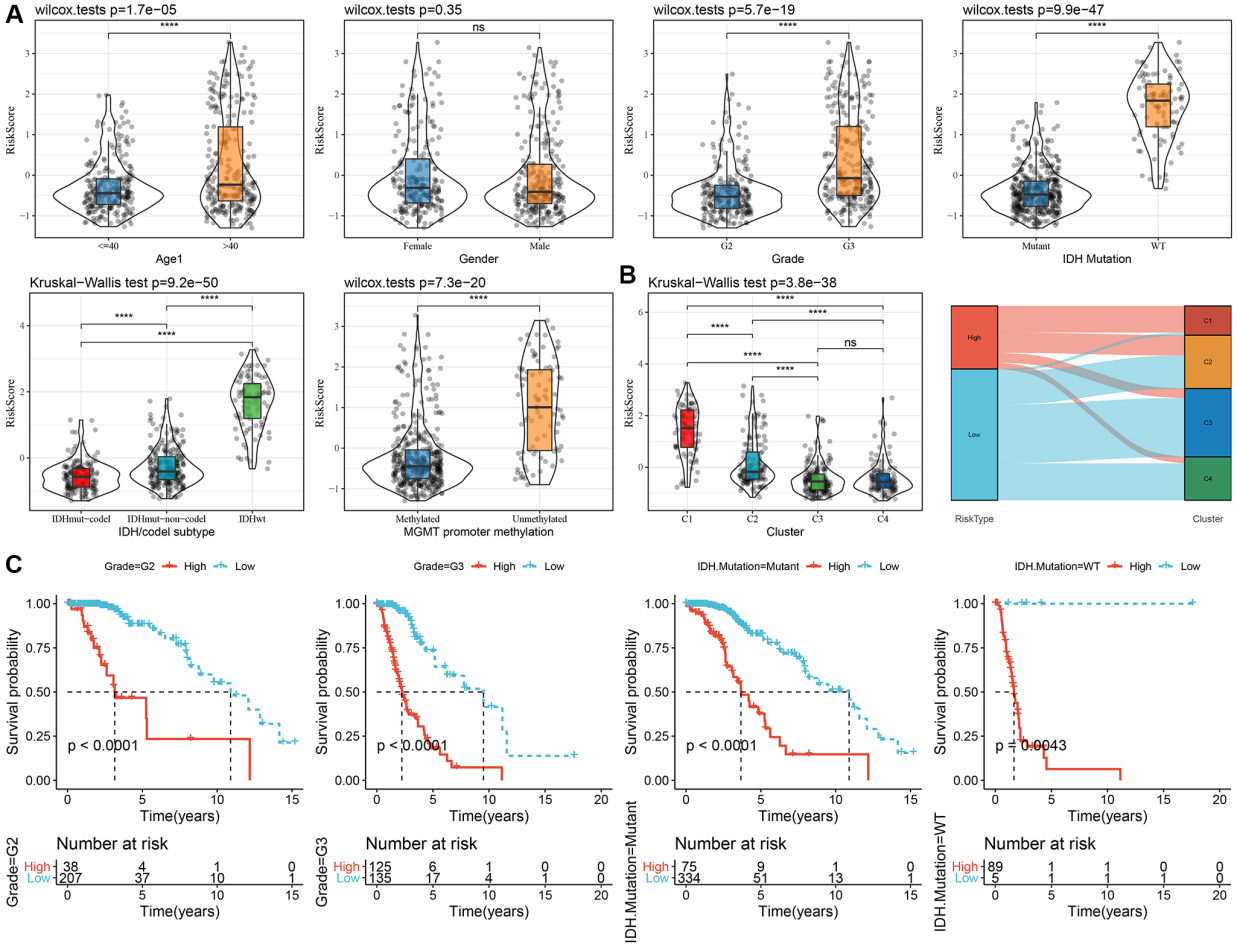
**Association of RiskScore with clinical information in TCGA dataset.** (**A**) Differences in RiskScore among different clinicopathological groups in the TCGA-LGG cohort; (**B**) Differences in RiskScore among different molecular subtypes in the TCGA-LGG cohort; (**C**) KM curves between high and low RiskScore groups in different clinical subgroup. Abbreviations: TCGA: The Cancer Genome Atlas; LGG: low-grade glioma; K-M: Kaplan-Meier.

To examine the relationship between the RiskScore and clinical features of LGG, we analyzed the differences in RiskScores across age, gender, grade, IDH mutation, IDH/code subtype, and MGMT promoter methylation in the TCGA-LGG dataset. Results showed that higher RiskScores were associated with increased age and disease grade (Figure 6A). Furthermore, we compared RiskScores among different molecular subtypes (Figure 6B) and found that the C1 subtype had the highest RiskScores. In addition, we also compared whether there are prognostic differences in the high- and low-risk groups of RiskScores defined here among different clinicopathological characteristic groups in the TCGA-LGG cohort. Results showed that our risk groups also performed well in different clinical groups, validating the reliability of our defined risk groups (Figure 6C). The findings in the CGGA dataset showed similar results (Supplementary Figure 5).

### Tumor immune microenvironment and molecular characteristics in both RiskScore groups

**Figure 7:**
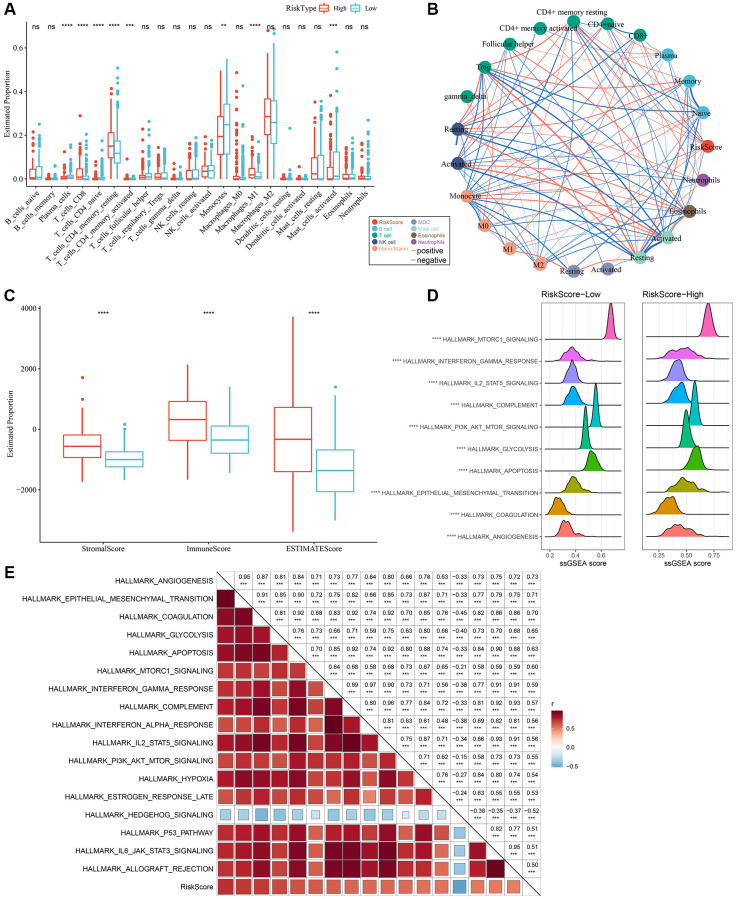
**Different infiltration levels of various immune cells between the two RiskScore groups.** (**A**) The amount of immune cell components in the TCGA cohort. (**B**) Correlation analysis of RiskScore with 22 immune cell components in the TCGA cohort. (**C**) The proportion of immune cell components in the TCGA cohort calculated by ESTIMATE software. The red box indicates high-risk group, and blue box indicates low-risk group. (**D**) Top 10 pathways with the most significant differences between low-RiskScore and high-RiskScore groups. (**E**) Correlation between KEGG pathways with correlation coefficient >0.5 and RiskScore. ^**^P < 0.01; ^***^P < 0.001; ^****^P < 0.0001. Abbreviations: ns: no significance; NK: natural killer; MDC: myeloid dendritic cell; ssGSEA: single-sample GSEA; GSEA: gene set enrichment analysis; TCGA: The Cancer Genome Atlas; KEGG: Kyoto Encyclopedia of Genes and Genome.

By employing the “CIBERSORT” algorithm, variation in the immune microenvironment of patients in both RiskScore groups was investigated by calculating the relative abundance of 22 immune cells. Of note, infiltration abundance of eight immune cells, including CD8^+^T cells, was found to be significantly different between the two groups (Figure 7A). Results of the correlation analysis demonstrated that RiskScores were closely related to immunocyte infiltration (Figure 7B). In addition, we also used ESTIMATE to evaluate immune cell infiltration, as shown in Figure 7C. We observed that the “ImmuneScore” in the “High” group was significantly higher than that in the “Low” group, and that LGG patients with a high RiskScore had higher immune cell infiltration.

In order to explore discrepancies in the molecular characteristics between high- and low-RiskScore groups, the ssGSEA algorithm was utilized to evaluate the activity of signaling pathways. As shown in Figure 7D, the activity of the following pathways in the high-RiskScore group were noticeably higher than those in the low-RiskScore group: interferon, glycolysis, apoptosis, and angiogenesis pathways, among others. Correlation analysis further validated the close association of the RiskScore with the above pathways (Figure 7E). Hence, dysfunction of the immune microenvironment and molecular signaling might be responsible for the different prognoses observed between high- and low-RiskScore groups.

### Chemotherapy and immunotherapy efficacy differences across RiskScore groups

**Figure 8:**
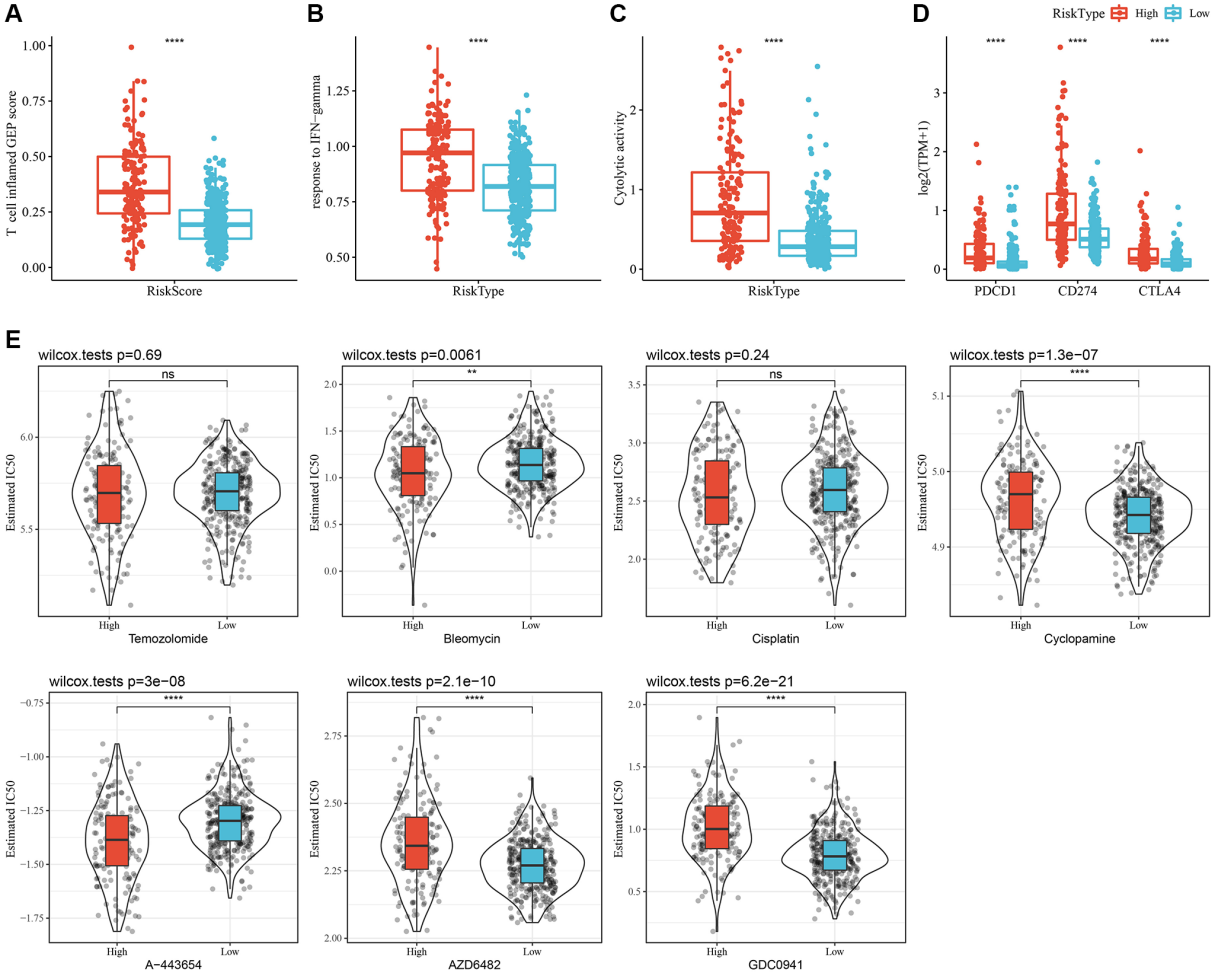
**Immunological characteristics and drug sensitivities between different RiskScore groups.** (**A**) The difference in “T cell inflamed GEP score” among various molecular subtypes. (**B**) The difference in “response to IFN-γ” among various molecular subtypes. (**C**) The difference in “Cytolytic activity” among various molecular subtypes. (**D**) Expression differences of immune checkpoint-associated genes among various molecular subtypes. (**E**) A box plot of the estimated IC_50_ values for temozolomide, bleomycin, cisplatin, cyclopamine, A-443654, AZD6482, and GDC0941 in TCGA-LGG. ^**^P < 0.01; ^****^P < 0.0001. Abbreviations: ns: no significance; GEP: gene expression profile; IFN: interferon; TPM: transcripts per million; IC_50_: half-maximal inhibitory concentration; TCGA: The Cancer Genome Atlas; LGG: low-grade glioma.

Initially, the “T-cell-inflamed GEP score” was used to assess the predictive potential of the RiskScore in ICB across different groups. The results suggested that, in the high-RiskScore group, the “T-cell-inflamed GEP score” and the “IFN-γ response” showed a considerable increase, and that the CYT score was also higher (Figure 8A–8C). Moreover, the expression of some checkpoint molecules (CTLA-4, PD-1, PD-L1) was noticeably higher (Figure 8D). Overall, patients with a high-RiskScore are likely to be more sensitivity to ICB.

In addition, patients with a high-RiskScore had lower IC_50_ values for bleomycin and A-443654, whereas patients with low-RiskScore had lower IC_50_ values for cyclopamine, AZD6482, and GDC0941. These findings suggested that high-RiskScore patients might benefit from treatment with bleomycin and A-443654; while low-RiskScore patients might benefit from cyclopamine, AZD6482, and GDC0941 (Figure 8E).

### RiskScore incorporates clinicopathological features to improve the prognosis models and survival prediction

**Figure 9:**
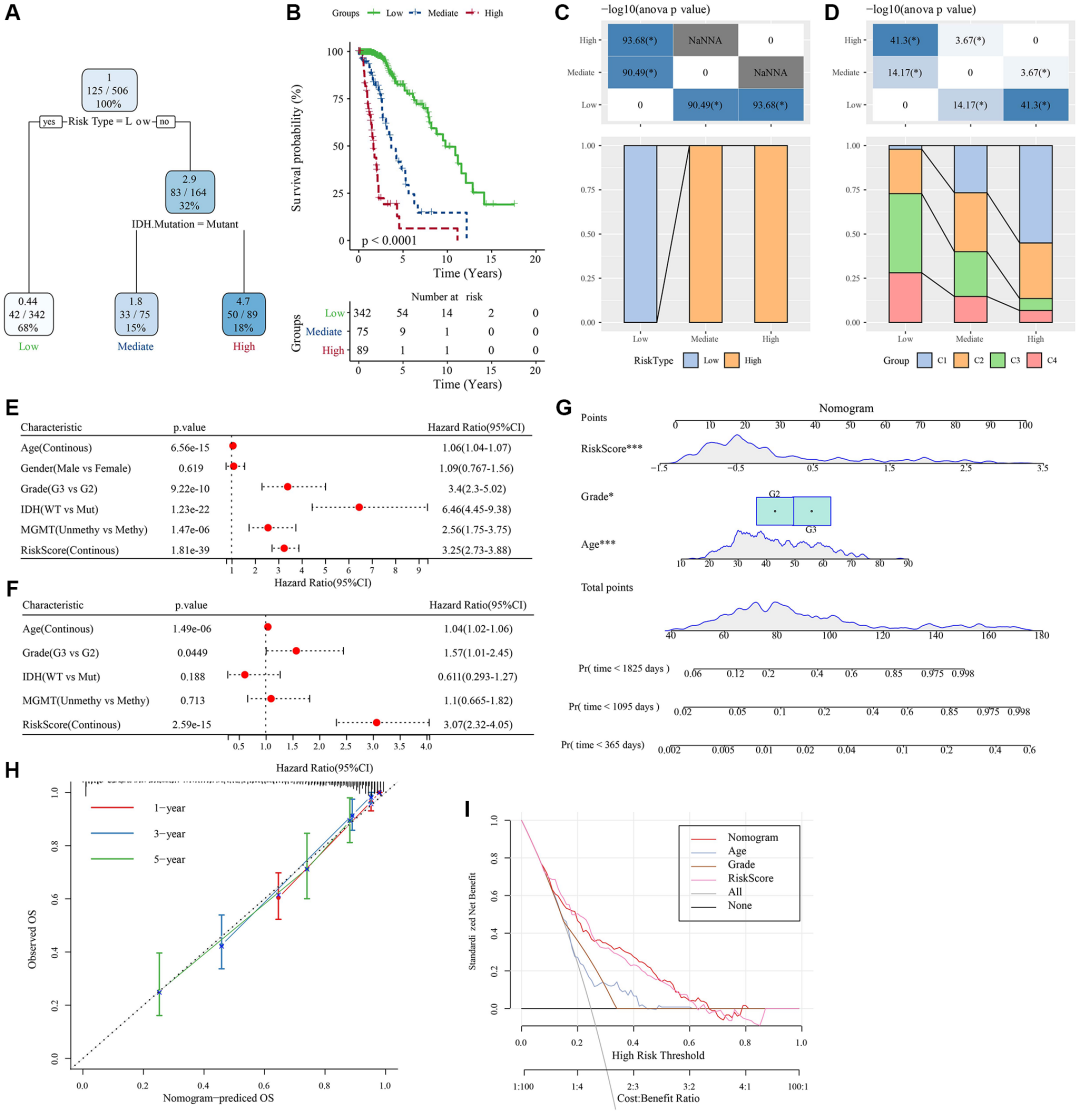
**Determination of optimal prognostic factors by decision tree and their reliability.** (**A**) Patients with full-scale annotations, including gender, RiskScore, age, and TNM stage, were employed to develop a survival decision tree for optimizing the risk stratification. (**B**) Significant differences in OS were found among the 4 risk subgroups. (**C**, **D**) Comparative analysis on different subgroups. (**E**, **F**) Univariate and multivariate Cox analyses of clinicopathological characteristics and RiskScore. (**G**) Nomogram model. (**H**) 1-, 3-, and 5-year calibration curves of the nomogram. (**I**) Decision curves of the nomogram. ^*^P < 0.05; ^***^P < 0.001. Abbreviations: ANOVA: analysis of variance; IDH: isocitrate dehydrogenase; WT: wild-type; Mut: mutant; MGMT: O-6-methylguanine-DNA methyltransferase; CI: confidence interval; OS: overall survival; TNM: tumor-node-metastasis; Pr: predicted.

To further improve the prognostic model, clinical information of patients (age, gender, tumor-node-metastasis (TNM) stage, *IDH* mutation, and RiskType) and RiskScores were integrated, and a decision tree model was constructed. Ultimately, only two parameters, *IDH* mutation and RiskType, were retained. Considering the maximum weight of RiskType, three subtypes were identified (Figure 9A) and their prognosis was examined (Figure 9B). The association of the decision tree model with the LASSO-Cox model is shown in Figure 9C, and the association of the decision tree model with consensus clusters is shown in Figure 9D. Univariate and multivariate Cox regression analyses highlighted the crucial role of the RiskScore in predicting clinical prognoses (Figure 9E, 9F). To quantify the risk assessment and survival probability of patients with LGG, we combined RiskScore and other clinicopathological features to establish a nomogram (Figure 9G). The calibration curve was used for model accuracy evaluation. The 1-, 3-, and 5-year predictive calibration curves nearly overlapped with the standard curve, suggesting a strong prediction performance (Figure 9H). In addition, the reliability of the model was assessed using the decision curve analysis (DCA). The RiskScore and nomogram benefits were both noticeably elevated when compared to the extreme curves, exhibiting strong survival prediction power over other clinicopathological characteristics (Figure 9I).

### Pan-cancer characterization of NK cell-related genes

**Figure 10:**
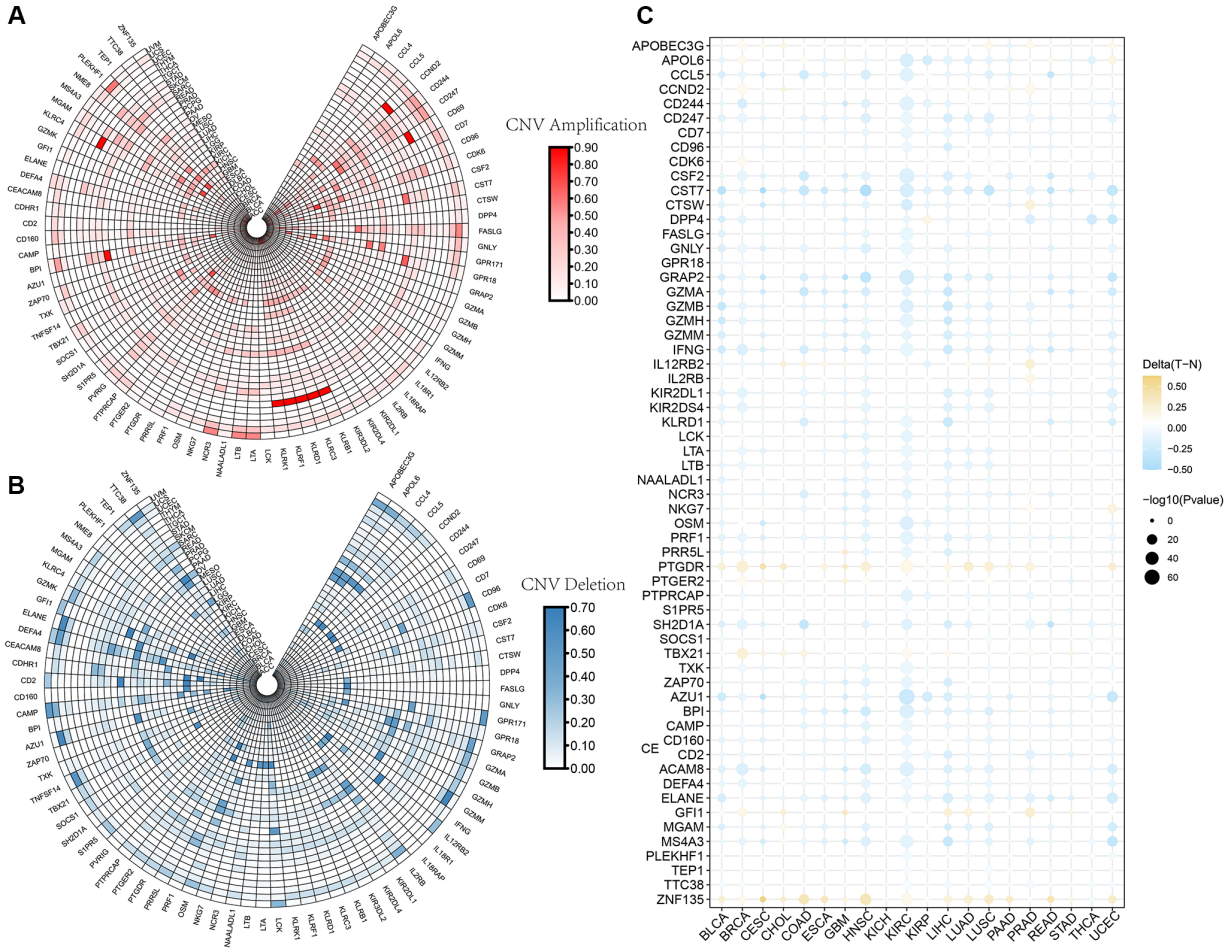
**The CNV and methylation profiles of NK cell-related genes in pan-cancer.** (**A**) CNV amplification of NK cell-related genes. (**B**) CNV deletion of NK cell-related genes. (**C**) DNA methylation traits of NK cell-related genes.

**Figure 11:**
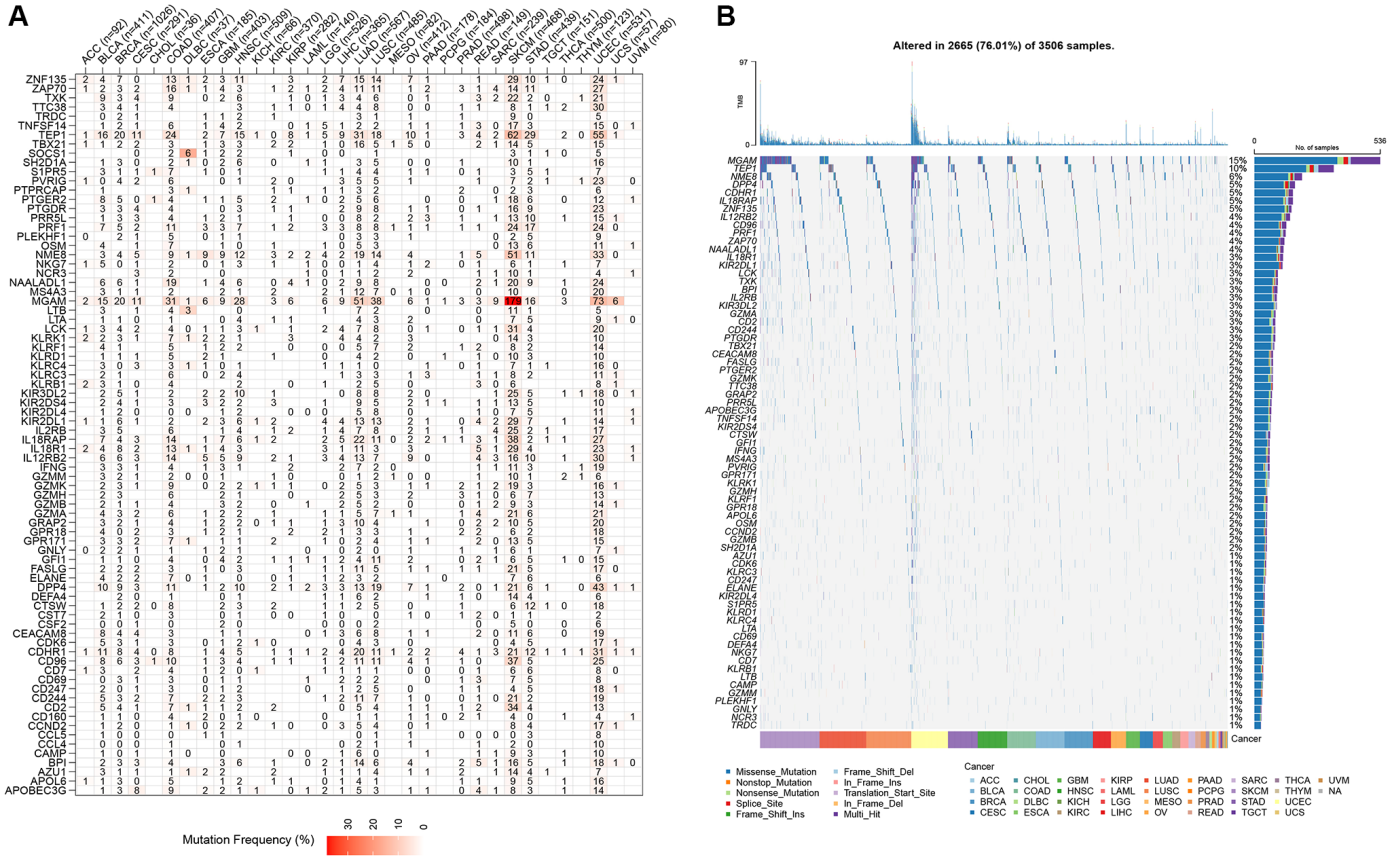
**The SNV profiles of NK cell-related genes in pan-cancer.** (**A**) Mutation frequency of NK cell-related genes in pan-cancer. (**B**) SNV oncoplot of NK cell-related genes.

**Figure 12:**
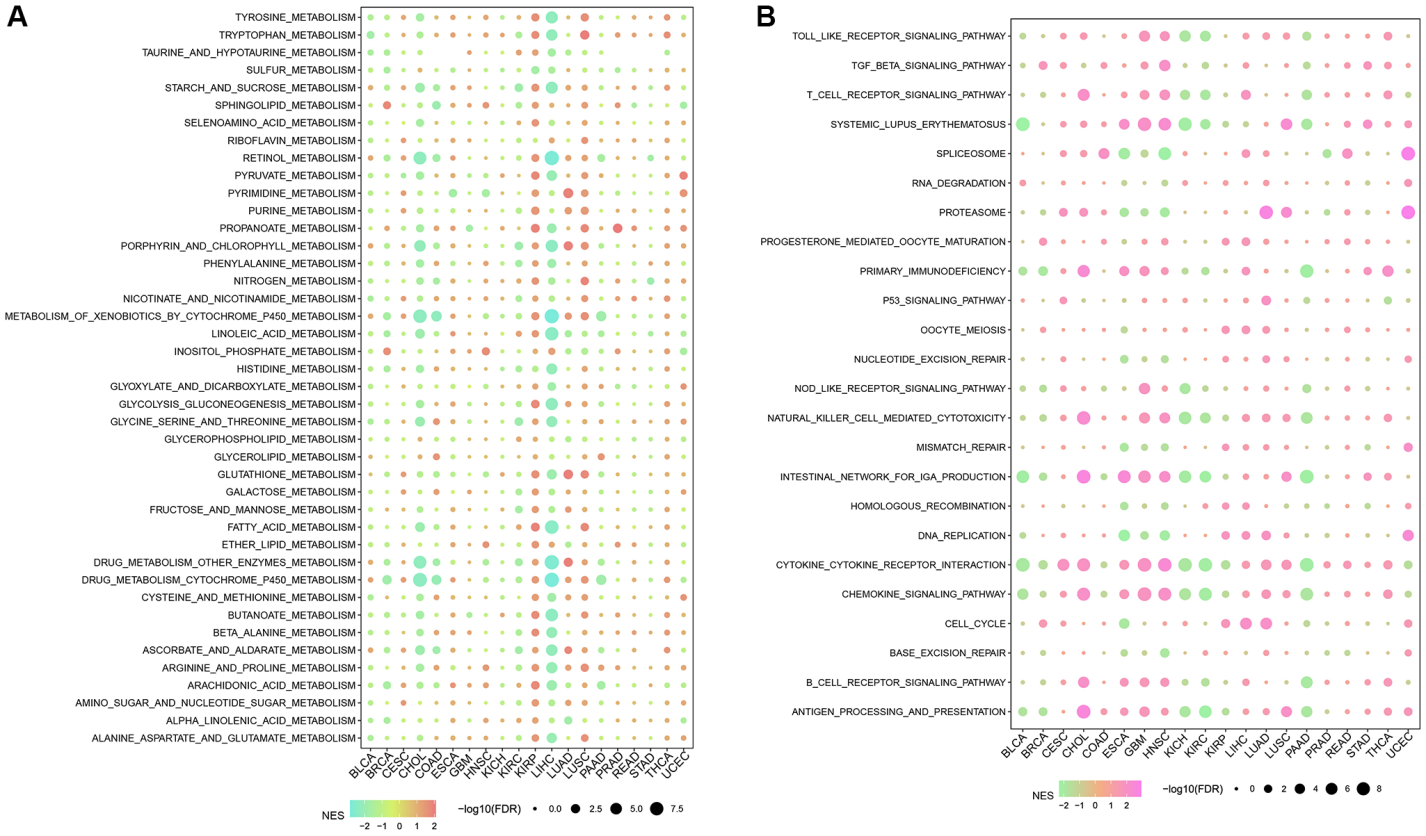
**The correlation among NK cell-related genes, tumor metabolic reprogramming, and immune microenvironment in pan-cancer.** (**A**) Correlation between NK cell-related genes and tumor metabolism-related pathways (**B**) Correlation between NK cell-related genes and immune-related pathways.

**Figure 13:**
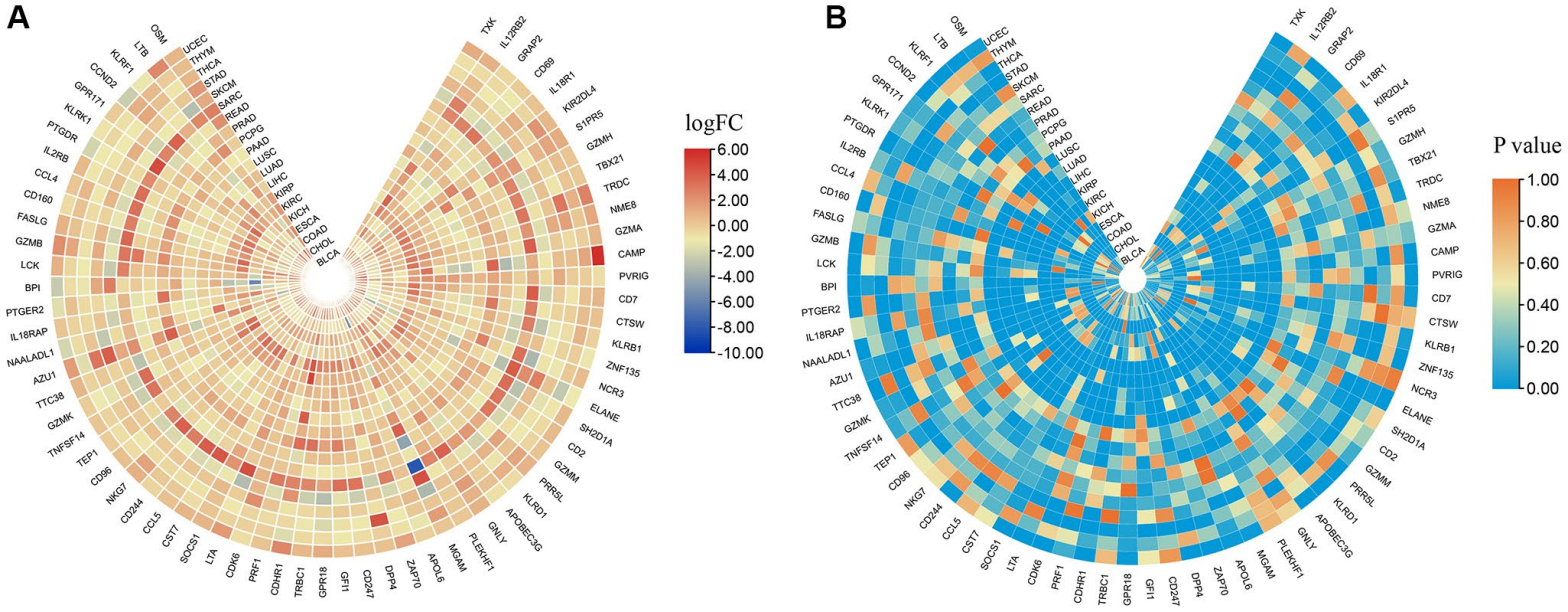
**The expression levels of NK cell-related genes in pan-cancer.** (**A**) logFC values of differential expression analysis. (**B**) *p* values of differential expression analysis.

The CNV profiles of NK cell-related genes in the pan-cancer cohort were summarized and plotted in Figure 10A, 10B. KLRK1, KLRF1, KLRD1, KLRC3, and KLRB1 showed relatively noticeable CNV amplification; while, GZMM, GZMH, GZMB, GZMA, and GRAP2 showed CNV deletion (Figure 10A, 10B). The majority of NK cell-related genes were accompanied by low methylation levels in cancers when compared to precancerous tissues. Of note, the methylation levels of PTGDR and ZNF135 genes were substantially higher in cancers when compared to precancerous tissues (Figure 10C). In addition, we also investigated other genomics traits (i.e., SNV). Results showed that mutation frequencies of MGAM and TEP1 were relatively high among all NK cell-related genes (Figure 11A, 11B). Importantly, we explored the correlation among NK cell-related genes, the tumor immune microenvironment, and tumor metabolic remodeling (Figure 12A, 12B). Generally, this regulatory network is rather complicated. Several cancer types showed distinct immunological and metabolic regulatory patterns for these NK cell-related genes, demonstrating the disease’s uniqueness among various tumor types. As for CHOL and LIHC, enrichment scores of NK cell-related genes were negatively correlated with tumor metabolic remodeling and positively correlated with the tumor immune microenvironment (Figure 12A, 12B). Finally, transcriptomic traits of NK cell-related genes in the pan-cancer cohort were also investigated (Figure 13A, 13B).

## DISCUSSION

Glioma, the most prevalent brain tumor in adults, accounts for approximately 81% of all brain tumors and has a poor prognosis; most LGGs progress to HGGs, making it a substantial area of clinical concern [40, 41]. The onset and progression of LGG is a multistep cascade process involving multiple factors such as genetics, gene mutations, cellular molecules, and immune dysregulation [42–44]. According to preclinical studies, glioma cells generate a significant variety of growth factors, cytokines, and chemokines that encourage the infiltration of many cells, including endothelial cells, pericytes, circulating progenitor cells, astrocytes, and certain immune cells [45–47]. In recent years, therapies targeting immune cell checkpoints have undergone remarkable advancement and achieved satisfactory clinical results in solid tumors. However, ICB is often ineffective in LGG due to the composition of the TME and the presence of immunosuppression. Therefore, an in-depth study of the immune composition of LGG is crucial to improve treatment stratification and associated patient prognoses.

NK cells (CD3^−^CD56^+^CD16^+^), potent cytotoxic lymphocytes that secrete perforin and granzyme, can kill cancer cells and virus-infected cells. Human leukocyte antigen (HLA) class I antigens interact with specific NK cell receptors to activate NK cells [48]. The main hurdle for NK cells to effectively kill glioma cells is the high expression of HLA class I. The *in vivo* disruption of immune homeostasis causes the reduction of HLA class I expression, thereby impairing NK cell tolerance [49]. Aberrant NK cell expression may cause glioma cells to escape immune surveillance. In the present study, differences in gene expression among different LGG subtypes were determined using NK cell-related genes. The findings of the enrichment analysis corroborated those of prior studies showing substantial differences between subtypes in terms of GEPs and associated pathways (mainly cell cycle and EMT-related pathways) [50]. Based on these findings, NK cells may contribute to LGG via both of these mechanisms. Moreover, the pan-characteristic of NK cell-related genes presented a wide range of CNVs and mutation statuses, confirming that NK cell gene mutations also contribute to the pathogenic mechanisms of tumor immune escape [51].

T cells are the primary immune cells targeted in ICB, and the balance between their activating and inhibiting signals plays a crucial role in ICB [52]. CTLA-4 and PD-1 are the two primary immunological checkpoint molecules on the surface of T cells. CTLA-4 modulates T cell activation during the first phase of immunological activation, while PD-1 is activated throughout the immune effector phase and is abundantly produced when tumor antigens are presented [53, 54]. In the present study, the expression of representative immune checkpoint molecules was evaluated in different LGG subtypes wherein *PD-1*, *PD-L1*, and *CTLA-4* were considerably highly expressed in the C1 subtype, which was consistent with a study by Ghouzlani et al. [55]. Therefore, patients in the C1 subtype may be potential candidates for ICB.

Most clinical trials investigating the use of *CTLA-4* and *PD-1* inhibitors in glioma are currently ongoing, with only a few published findings, and none achieving significant efficacy [56–58]. The reason for this may be attributed to drug resistance. There are many mechanisms associated with the resistance of ICB in the treatment of glioma, which can be broadly categorized as endogenous or exogenous. Endogenous mechanisms include alterations in immune recognition sites, abnormal cellular signaling pathways, alterations in gene expression, and DNA damage repair [59]. On the other hand, exogenous mechanisms include all factors related to immune cell activation [60]. Common factors used to determine patient prognoses and prediction of treatment outcomes include *PD-1*/*PD-L1* expression levels, TMB, tumor-infiltrating lymphocytes (TILs), and microsatellite instability (MSI) [61]. In the present study, ssGSEA revealed that *PD-1*/*PD-L1*, TMB, and TILs were significantly different among LGG subtypes, highlighting the diversity of gliomas and providing new indicators for predicting ICB efficacy.

Construction of a risk model enables the more exact identification of high-risk groups. In the present study, a prediction model was successfully constructed based on 6 NK cell-related genes, and patients were classified into high- and low-risk groups. Prostaglandin F2 receptor negative regulator (*PTGFRN*), a tumorigenesis-related gene, was associated with interleukin (IL)-12-mediated tumor recognition and killing efficacy for drugs [62, 63]. The role of *HSPB1* in tumor immunity has been previously demonstrated, and its mechanism of action is related to the direct immunosuppression of Ym1 produced by macrophages and T cell suppression [63]. The sterile alpha motif domain-containing protein 9 (*SAMD9*) is a potential antigen for the development of messenger RNA (mRNA) vaccines against diffuse glioma. Its expression is associated with tumor immune subtypes and determines immune-related processes of tumor-associated genes [64]. In addition, the expression of secreted frizzled-related protein 2 (*SFRP2*), a member of the secretory glycoprotein family, correlates with the degree of immune infiltration of tumor cells and plays a synergistic role in tumor progression. Therefore, *SFRP2* is a promising prognostic biomarker and therapeutic target [65]. Although less research has been done on elastin Microfibril Interfacer 3 (*EMILIN3*) and cartilage acidic protein 1 (*CRTAC1*) in tumor immunity, previous findings suggest that *CRTAC1* may influence the onset and progression of tumors by binding to calcium ions and innate immune pathways [66].

Given the poor prognosis of individuals with LGG, it is vital to discover clinical variables that influence their prognosis and to implement appropriate therapies. This research successfully divided LGG patients into two groups by calculating RiskScores and determining patient prognoses, which were found to be significantly different. In addition, a nomogram was constructed and validated to determine the ability to accurately predict the prognosis of patients with LGG. Overall, the nomogram exhibited good prognostic ability and was a powerful tool in predicting the survival status of individuals with LGG across clinical settings. This study is the first to identify molecular subtypes and prognostic models associated with LGG based on NK cells. In addition, this new six-gene prognostic model has not been previously reported. Nonetheless, there are some limitations to this study, including the validation of these findings using PCR or immunohistochemical experiments. Moreover, some clinical factors were not considered due to the lack of necessary clinical follow-up information, especially diagnostic details. These limitations may influence the effectiveness of the clinical application of our RiskScore.

## CONCLUSIONS

In this study, molecular subtypes were categorized using consistent clustering based on NK cell-correlated genes and showed different immunological, pathological, prognostic, and pathway features. Subsequently, from these molecular subtypes, 6 key NK cell-related genes were screened based on DEGs, and a clinical prognostic model was developed. This model was independent of other clinicopathological features, demonstrating stable predictive performance in independent datasets and strong robustness. We finally integrated clinicopathological characteristics with RiskScores and constructed a decision tree model to further improve its survival prediction. Overall, this tool may be used for patient stratification, particularly for ICB. Moreover, our study may provide critical information to overcome immune resistance by targeting NK cell-related genes.

## Supplementary Materials

Supplementary Figures

Supplementary Table 1

Supplementary Tables 2-5
