# Supplementary material for: Identification of natural killer cell-related characteristics to predict the clinical prognosis and immune microenvironment of patients with low-grade glioma
Source: Aging (Albany NY). 2023 Jul 5;15(13):6264–91. doi: 10.18632/aging.204850 (PMC10373982; doi:10.18632/aging.204850)
Supplement: Supplementary Figures [file aging-15-204850-s001.pdf]

SUPPLEMENTARY FIGURES

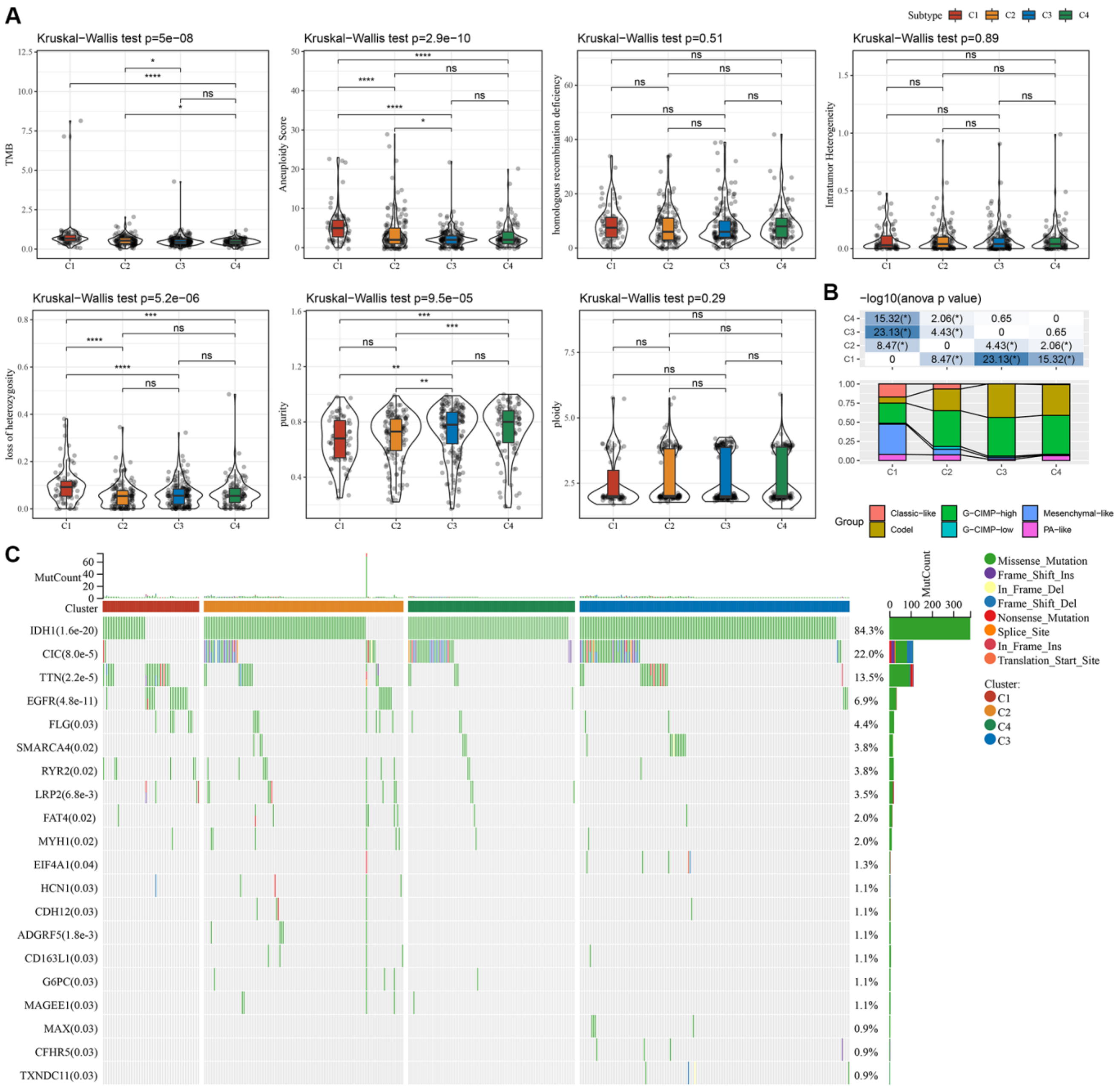

Supplementary Figure 1. Mutation burden in different molecular subtypes. (**A**) Comparison of TMB, aneuploidy score, homologous recombination defects, intratumor heterogeneity, LOH, purity, and ploidy among different molecular subtypes in the TCGA-LGG cohort. (**B**) Comparison of the four molecular subtypes with immune molecular subtypes. (**C**) Somatic mutations in the four molecular subtypes (chi-square test). $^{*}P < 0.05$. $^{**}P < 0.01$; $^{***}P < 0.001$; $^{****}P < 0.0001$. Abbreviations: ns: no significance: ANOVA; analysis of variance; IDH: isocitrate dehydrogenase; WT: wild-type; Mut: mutant; MGMT: O-6-methylguanine-DNA methyltransferase; TMB: tumor mutation burden; LOH: loss of heterozygosity; TCGA: The Cancer Genome Atlas; LGG: low-grade glioma.

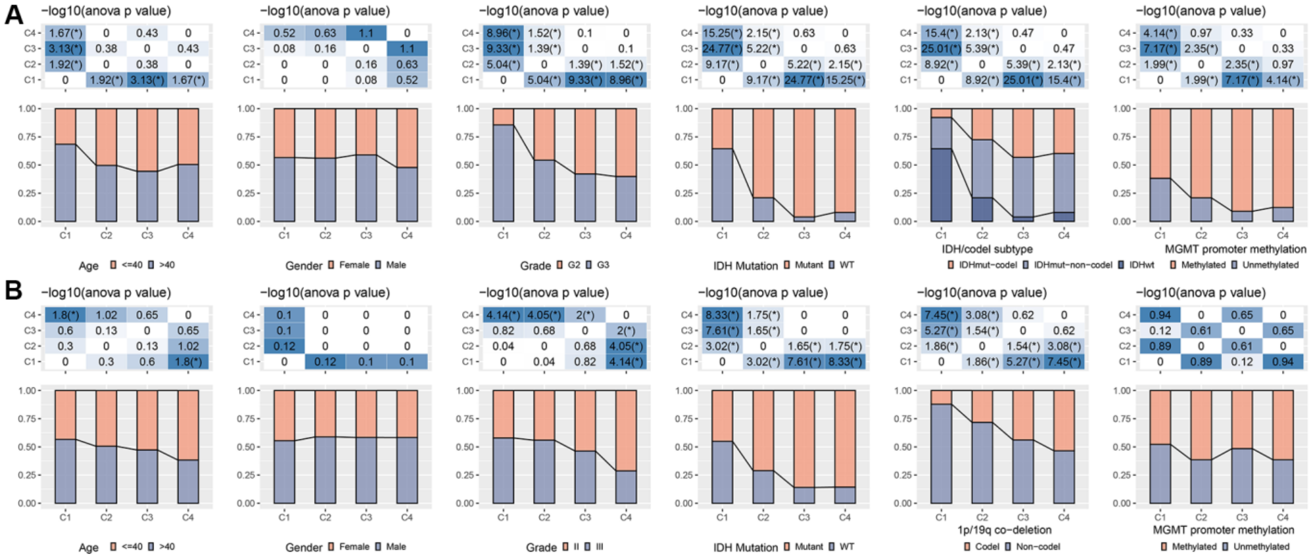

Supplementary Figure 2. Distribution patterns of various molecular subtypes in each clinical variable. (**A**) Clinicopathological characteristics of molecular subtypes in the TCGA-LGG cohort. (**B**) Clinicopathological characteristics of molecular subtypes in the CGGA cohort; the upper part is the statistical significance of the pairwise distribution difference, the lower part is the proportion. Abbreviations: TMB: tumor mutation burden; ANOVA: analysis of variance; TCGA: The Cancer Genome Atlas; LGG: low-grade glioma; CGGA: Chinese Glioma Genome Atlas.

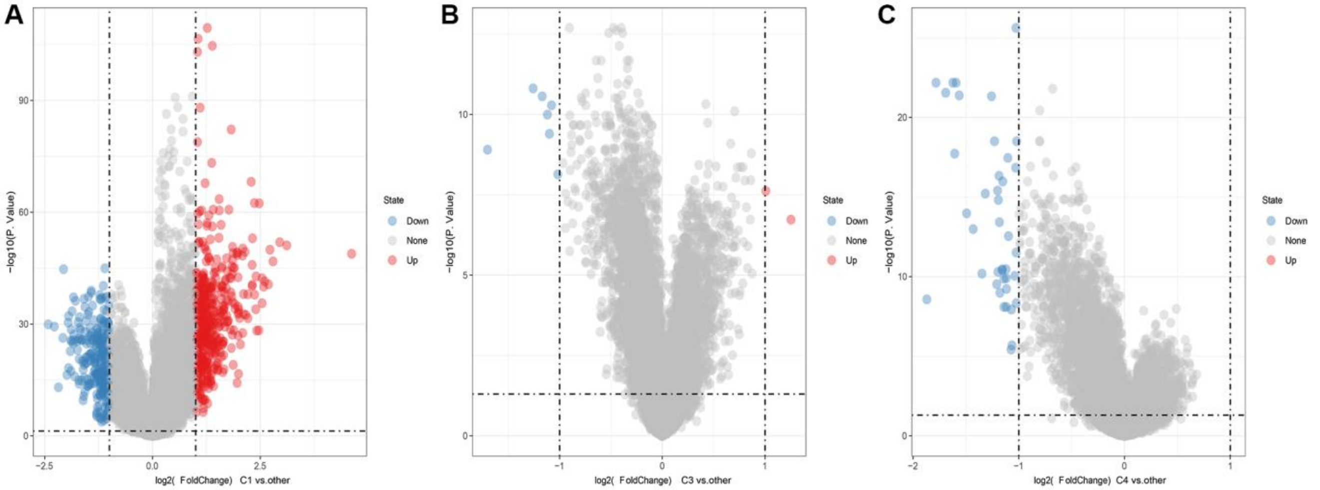

Supplementary Figure 3. Identification of DEGs among different clusters. (**A**) C1 vs. other. (**B**) C3 vs. other. (**C**) C4 vs. other. Abbreviation: DEGs: Differentially expressed genes.

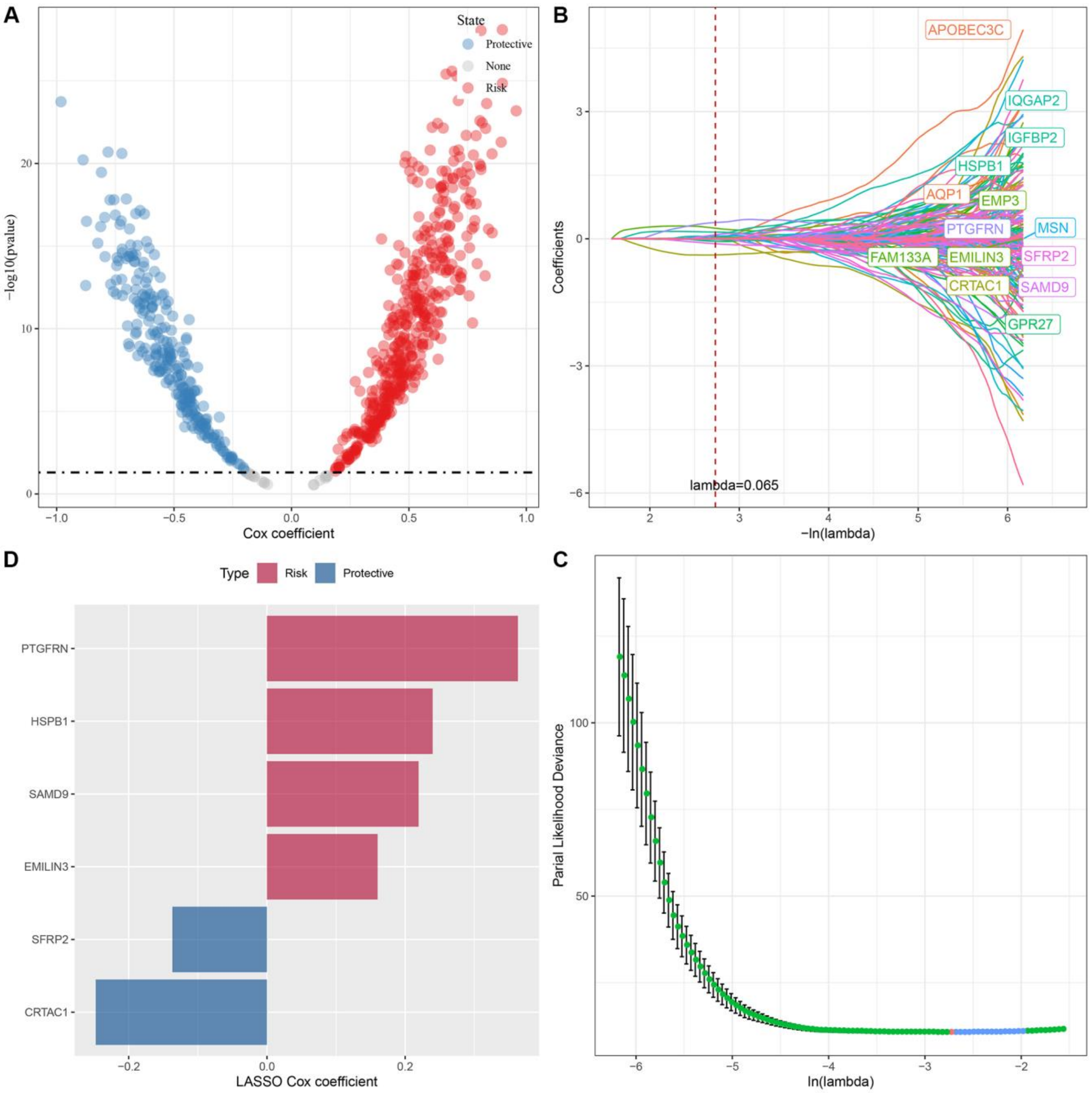

Supplementary Figure 4. Construction of a prognostic model with key genes screened by LASSO algorithm. (**A**) A total of 719 promising candidates were filtered from all the DEGs. (**B**) Changed trajectory of each independent variable with lambda. (**C**) Confidence interval under lambda. (**D**) Distribution of LASSO-Cox coefficients of the NK cell-associated prognostic gene signature. Abbreviations: LASSO: least absolute shrinkage and selection operator; DEGs: differentially expressed genes; NK: natural killer.

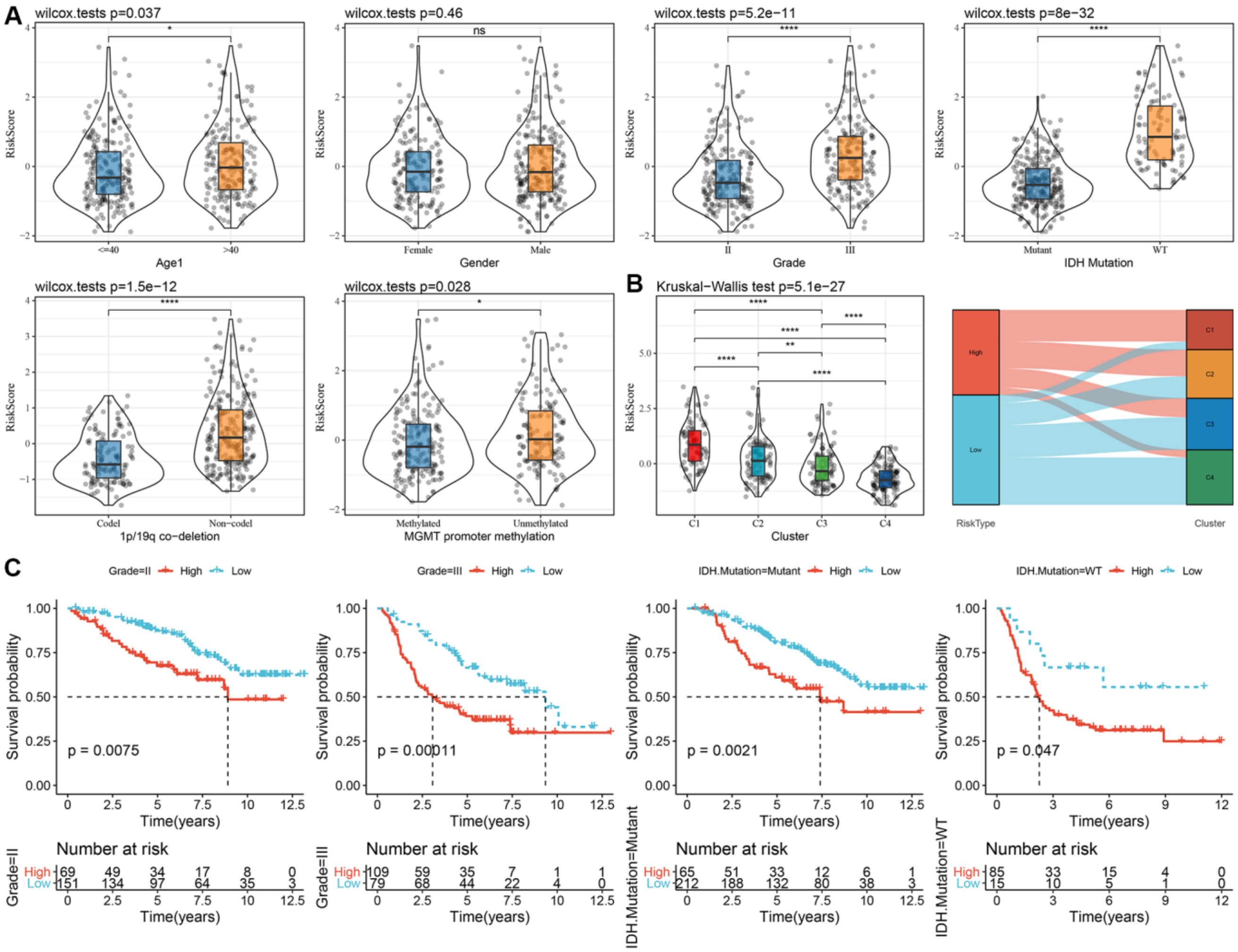

Supplementary Figure 5. Association of RiskScore with clinical information in CGGA dataset. (**A**) Differences in RiskScore among different clinicopathological groups in the CGGA-LGG cohort. (**B**) Differences in RiskScore among different molecular subtypes in the CGGA-LGG cohort. (**C**) KM curves between high and low RiskScore groups in different clinical subgroup. Abbreviations: CGGA: Chinese Glioma Genome Atlas; LGG: low-grade glioma; K-M: Kaplan-Meier.
