# Supplementary material for: Identification of natural killer cell-related characteristics to predict the clinical prognosis and immune microenvironment of patients with low-grade glioma
Source: Aging (Albany NY). 2023 Jul 5;15(13):6264–91. doi: 10.18632/aging.204850 (PMC10373982; doi:10.18632/aging.204850)
Supplement: Supplementary Table 1 [file aging-15-204850-s002.pdf]

SUPPLEMENTARY TABLES

Supplementary Table 1. The clinical information of TCGA and CGGA dataset.

Characteristics	TCGA (*N* = 506)	CGGA (*N* = 408)	Total (*N* = 914)
Age			
≤40	249 (27.24%)	214 (23.41%)	463 (50.66%)
>40	257 (28.12%)	193 (21.12%)	450 (49.23%)
NA	0 (0.0e + 0%)	1 (0.11%)	1 (0.11%)
Gender			
Female	226 (24.73%)	172 (18.82%)	398 (43.54%)
Male	280 (30.63%)	236 (25.82%)	516 (56.46%)
Grade			
G2	245 (26.81%)	220 (24.07%)	465 (50.88%)
G3	260 (28.45%)	188 (20.57%)	448 (49.02%)
NA	1 (0.11%)	0 (0.0e + 0%)	1 (0.11%)
IDH.Mutation			
Mutant	409 (44.75%)	277 (30.31%)	686 (75.05%)
WT	94 (10.28%)	100 (10.94%)	194 (21.23%)
NA	3 (0.33%)	31 (3.39%)	34 (3.72%)
IDH.codel.subtype			
IDHmut-codel	165 (18.05%)	0 (0.0e + 0%)	165 (18.05%)
IDHmut-non-codel	244 (26.70%)	0 (0.0e + 0%)	244 (26.70%)
IDHwt	94 (10.28%)	0 (0.0e + 0%)	94 (10.28%)
NA	3 (0.33%)	408 (44.64%)	411 (44.97%)
MGMT.promoter.methylation			
Methylated	418 (45.73%)	191 (20.90%)	609 (66.63%)
Unmethylated	88 (9.63%)	150 (16.41%)	238 (26.04%)
NA	0 (0.0e + 0%)	67 (7.33%)	67 (7.33%)
1p.19q.co. deletion			
Codel	0 (0.0e + 0%)	131 (14.33%)	131 (14.33%)
Non-codel	0 (0.0e + 0%)	243 (26.59%)	243 (26.59%)
NA	506 (55.36%)	34 (3.72%)	540 (59.08%)
